# Evolution of the *Perlecan/HSPG2* Gene and Its Activation in Regenerating *Nematostella vectensis*

**DOI:** 10.1371/journal.pone.0124578

**Published:** 2015-04-15

**Authors:** Curtis R. Warren, Elias Kassir, James Spurlin, Jerahme Martinez, Nicholas H. Putnam, Mary C. Farach-Carson

**Affiliations:** 1 Department of BioSciences, Rice University, Houston, Texas, United States; 2 Department of Bioengineering, Rice University, Houston, Texas, United States; UC Irvine, UNITED STATES

## Abstract

The *heparan sulfate proteoglycan 2 (HSPG2)/perlecan* gene is ancient and conserved in all triploblastic species. Its presence maintains critical cell boundaries in tissue and its large (up to ~900 kDa) modular structure has prompted speculation about the evolutionary origin of the gene. The gene’s conservation amongst basal metazoans is unclear. After the recent sequencing of their genomes, the cnidarian *Nematostella vectensis* and the placozoan *Trichoplax adhaerens* have become favorite models for studying tissue regeneration and the evolution of multicellularity. More ancient basal metazoan phyla include the poriferan and ctenophore, whose evolutionary relationship has been clarified recently. Our *in silico* and PCR-based methods indicate that the *HSPG2* gene is conserved in both the placozoan and cnidarian genomes, but not in those of the ctenophores and only partly in poriferan genomes. *HSPG2* also is absent from published ctenophore and *Capsaspora owczarzaki* genomes. The gene in *T*. *adhaerens* is encoded as two separate but genetically juxtaposed genes that house all of the constituent pieces of the mammalian *HSPG2* gene in tandem. These genetic constituents are found in isolated genes of various poriferan species, indicating a possible intronic recombinatory mechanism for assembly of the *HSPG2* gene. Perlecan’s expression during wound healing and boundary formation is conserved, as expression of the gene was activated during tissue regeneration and reformation of the basement membrane of *N*. *vectensis*. These data indicate that the complex *HSPG2* gene evolved concurrently in a common ancestor of placozoans, cnidarians and bilaterians, likely along with the development of differentiated cell types separated by acellular matrices, and is activated to reestablish these tissue borders during wound healing.

## Introduction

The mammalian *HSPG2/perlecan* gene product has dual functional roles in maintaining tissue boundaries and providing a growth factor depot for wound healing [1]. The modern gene *HSPG2* encodes perlecan, an extracellular heparan sulfate proteoglycan of high (up to ~900 kDa) molecular weight and composed of 48 modular units sharing homology with other extracellular matrix (ECM) proteins [2]. The human core protein consists of 4,370 amino acids and is modified with 3–4 heparan or chondroitin sulfate chains before secretion into the extracellular space, requiring a costly metabolic investment by the cell manufacturing it. Perlecan performs signaling, adhesive and extracellular scaffolding roles [3], such that mutation of the gene has pleiotropic effects; complete loss of the perlecan gene is lethal in humans and mice [4]. *HSPG2* orthologues have been studied in vertebrates, arthropods, and nematodes [5,6]. A goal of this study was to use the publicly available genomes of the basal metazoans *Amphimedon queenslandica* [7] and other porifera, *Trichoplax adhaerens* [8] and the cnidarian *Nematostella vectensis* [9] as well as the ctenophore *Mnemiopsis leidyi* [10] and the choanoflagellate *Monosiga brevicollis* [11] to infer the evolutionary events that created this large, complex, highly conserved proteoglycan.

Placozoans are small (1-3mm), morphologically simple animals. Genomic analyses suggest that placozoans are more closely related to cnidarians and bilaterians than sponges and ctenophores, however the evolutionary relationships among basal metazoan lineages continues to be disputed [12]. Choanoflagellates make up a clade of single-celled organisms closely related to metazoans. The recently sequenced genome of the choanoflagellate *M*. *brevicollis* encodes few ECM-like proteins [11,13]. Given the uncertainties in understanding the evolutionary relationships among these basal lineages, we hypothesized that the study of assembly of the complex *HSPG2* gene orthologues will offer unique insights into species relationships and early evolution of multicellularity in development and its re-establishment in wound healing.

The cnidarian *N*. *vectensis* is used as a model of tissue regeneration and of diploblastic development [14]. *N*. *vectensis* completely regenerate after lateral bisection, allowing visualization and molecular characterization of the regenerative process [15]. *HSPG2* expression is activated during wound healing in humans in the various stages of wound reconstruction [16]. In resting human tissue, perlecan is deposited into the basal lamina. The cnidarian ectoderm secretes a basal lamina ECM that separates epithelial and connective tissues[17]. To examine the potential for a conserved function of perlecan in tissue regeneration, we examined the presence of the *HSPG2* orthologue during regeneration of *N*. *vectensis*.

We demonstrate that *T*. *adhaerens perl* is encoded as two separate but clearly identifiable genes in the same region of the placozoan genome. We speculate on the evolution of the *perl* gene by identifying scattered constituents of the gene in poriferans, and its essential absence in the choanoflagellates and ctenophores. Consistent with a role in reestablishment of mesoglea in triploblastic species, we show re-expression during the regeneration of *N*. *vectensis* oral structures.

## Materials and Methods

### *Perl* gene Prediction

Genomic scaffold and transcript sequences were downloaded from the Joint Genome Institute (http://www.jgi.doe.gov/) [7,8,9,11]. tBLASTn was used to compare human perlecan peptide sequences to transcript sequences of the experimental animal. This approach identified genomic regions with high similarity to some portion of the human *HSPG2* gene. Independently folding protein modules of the human perlecan protein were aligned with all translated frames of scaffolds identified by tBLASTn analysis. *T*. *adhaerens* and *N*. *vectensis* genomic regions that aligned with a >20% peptide homology with human Ig modules or >30% peptide homology with all other human perlecan modules were inputted into Genewise WISE2 (http://www.ebi.ac.uk/Tools/psa/genewise/) to predict exon-intron boundaries of the putative *perl* genes. All sequence analysis was performed in using Geneious version 5.4 created by Biomatters.

### *Trichoplax adhaerens* Culture

*T*. *adhaerens* were fed on *Porphyridium* algae (Carolina Biological Supply—Burlington, NC) maintained in Artificial Seawater (ASW)—formulated from 35g of Reef Crystals Reef Salt (Foster and Smith, Rhinelander, WI) in 1L of ddH20. This ASW was supplemented with 1mL Micro Algae Grow (Florida Aqua Farms Inc,—City, St) to make seawater medium. *Porphyridium* subcultures were established in glass petri dishes one week prior to seeding with *T*. *adhaerens* organisms. Petri dishes with established *T*. *adhaerens* cultures had fresh ASW medium added every week. New *T*. *adhaerens* cultures were established every week.

### *Nematostella vectensis* Culture

*N*. *vectensis* were purchased from the Marine Biological Laboratory (Woods Hole, MA). The animals were maintained in ASW diluted 1:3 in ddH_2_0. They were fed *Artemia nauplii* (Aqua Medic, Loveland, CO) and their 1:3 ASW was refreshed at least once weekly. Regeneration assays were performed according to the published standard [18].

### Ribonucleic Acid Isolation

RNA was obtained by starving an established *T*. *adhaerens* culture overnight in Artificial Seawater (ASW) to remove the majority of the *Porphyridium*. These dishes were rinsed the following day in ASW, and TRIzol reagent (Life Technologies, Carlsbad, CA) was added directly to the dish. RNA was extracted following the manufacturer’s instructions. *N*. *vectensis* were starved for 3 days prior to RNA isolation. Animals were disrupted using a tissue homogenizer and RNA was extracted as published previously [19].

### 5′ Rapid Amplification of cDNA Ends (RACE)

RACE PCR reactions were conducted with the 5’ RACE System (Life Technologies—Carlsbad, CA) according to the manufacturer’s instructions. PCR products were subcloned and sequenced. The sequences of the *T*. *adhaerens* and *N*. *vectensis perl* genes are uploaded to genbank with the accession numbers KP995435 and KP995436 respectively.

### Phylogenetic Analysis of *Perl*

Briefly, the sequences for known perlecan genes were downloaded from NCBI or manually inserted based upon our sequencing and BLAST of the *N*. *vectensis* and *T*. *adhaerens* genes as well as the *perl II* homologue of *C*. *candelabrum*. The evolutionary history of perlecan was inferred by using the Maximum Likelihood method based on the Whelan And Goldman + Freq. model [20]. The tree with the highest log likelihood (-24153.6381) is shown. Initial tree(s) for the heuristic search were obtained by applying the Neighbor-Joining method to a matrix of pairwise distances estimated using a JTT model. A discrete Gamma distribution was used to model evolutionary rate differences among sites (5 categories (+*G*, parameter = 2.4818)). The tree is drawn to scale, with branch lengths measured in the number of substitutions per site. All positions with fewer than 95% site coverage were eliminated. That is, fewer than 5% alignment gaps, missing data, and ambiguous bases were allowed at any position. There were a total of 1451 positions in the final dataset. Evolutionary analyses were conducted in MEGA6 [21]. The figure was assembled using the Figtree phylogenetic tree production program (http://tree.bio.ed.ac.uk/software/).

### *In Situ* Hybridization of Sectioned *N*. *vectensis*

*N*. *vectensis* were starved for three days prior to fixation. In the case of regeneration studies, animals were starved three days prior to bisection. Animals were fixed in modified Carnoy’s solution as previously described [22], embedded in paraffin and sectioned longitudinally. *In situ* hybridization temperature was optimized at 63°C and was performed as previously described [23]. The primers for amplification of the riboprobe sequence were as follows: Forward primer (5′ to 3′): CGGCGGACAACTCCCCAAGG, Reverse Primer (5′ to 3′): GAGAACATTGCCCTCGATGAC, and are directed at sequences of *perl* domain IV. Two biological replicates were performed for each regenerative stage.

### Quantitative Polymerase Chain Reaction

RNA was collected from regenerating physa of *N*. *vectensis* polyps following a transverse bisection at the terminus of the mesenteries. Various time points were chosen for RNA collection (0, 2, 4, 6 and 8 days post bisection), coinciding with early stages of polyp regeneration as previously characterized [18]. A total of 3 polyps were used for each time point. Reverse transcription reactions were performed using the qScript cDNA Supermix kit (Quanta Biosciences, Gaithersburg, MD). Quantitative PCR was performed using B-R SYBR Green SuperMix for IQ following the manufacturer’s instructions (Quanta Biosciences, Gaithersburg, MD). The thermal cycling program was as follows: 95°C for 3 min, then 40 cycles of the following three steps: 95°C for 5 s, 60°C for 15 s, and 72°C for 15 s. *HSPG2* was normalized to the *GAPDH* transcript levels and relative mRNA levels were calculated using the2^−ΔΔCT^ method. *HSPG2* primers: Forward—GGCCCGGCGATCATCCGAAG and Reverse—GTAGGGTCCCCTCCGACTCC. *GAPDH* primers: Forward—GGACCAAGTGCCAAGAACTG and Reverse—GGAATGCCATACCCGTCAG.

## Results

### *HSPG2*-related *perl* is conserved in *N*. *vectensis and T*. *adhaerens*

**Fig 1:**
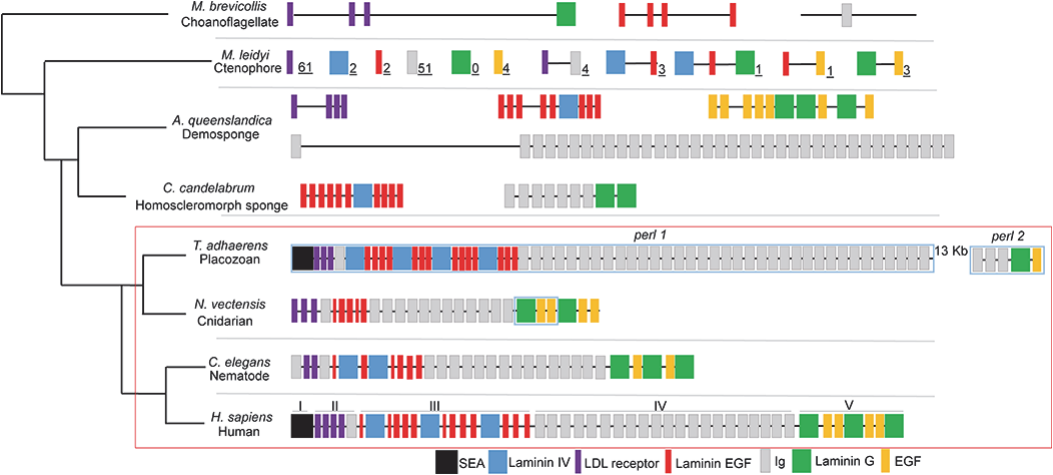
Predicted perlecan orthologues or constituents in model organisms. The presumptive pre-perlecan constituents of *A*. *queenslandica* (A) and perlecan proteins of *T*. *adhaerens*, *N*. *vectensis*, *C*. *elegans*, and *H*. *sapiens* are shown in schematic form to demonstrate structural differences. Several of the scarce matches for perlecan modules in the *M*. *brevicollis* and *A*. *queenslandica* genomes are shown in schematic form. The number of unique matches for individual or paired perlecan folding modules in the *M*. *leidyi* transcriptome is shown in subscript next to each schematic module. The double line between *T*. *adhaerens perl 1* and *T*. *adhaerens perl 2* indicates that these two genes, although only thirteen kb apart on the chromosome, are separately transcribed. Blue rectangles represent parts of the *T*. *adhaerens* and *N*. *vectensis* transcripts amplified by RACE PCR and sequenced; all other parts of the protein were predicted by tBLASTn-alignment. Schematics are not to scale; gene lengths are denoted at the right end of each diagram. The red dashed line encompasses the “perlecan” clade of the animal kingdom.Surprisingly, the easily recognizable *perl* gene was found in the placozoan *T*. *adhaerens* (Fig 1). All five protein domains are included in the *T*. *adhaerens perl* gene, in the identical order to that of *HSPG2* in humans. *T*. *adhaerens perl* domain I includes a SEA module, which, until now, was thought to be found exclusively in perlecan of mammals and birds. The *T*. *adhaerens perl* SEA module includes the G-SVV motif, critical to the autocleavage of these modules in other proteins. Whereas the SEA module of perlecan in mammals and birds does not contain this autocleavage motif, we speculate that this ancestral perlecan molecule may have undergone autocleavage while in the secretory pathway. *T*. *adhaerens perl* domain II is structured exactly as that of human *HSPG2*, with three LDL receptor-like repeats followed by one immunoglobulin (Ig) domain. Domain III of *T*. *adhaerens* perl is also similar to that of *H*. *sapiens* perlecan/*HSPG2*, however in comparison, *T*. *adhaerens* perl includes one additional set of Laminin B- and Laminin EGF-like modules. Domain IV of *T*. *adhaerens* perl encodes roughly 36 total Ig modules, making it substantially larger than the domain IV of human perlecan, which only includes 21 Ig modules. Domain V of *T*. *adhaerens* perl encodes both the laminin G and EGF-like modules similar to other perlecan proteins.

The *perl* gene (an *HSPG2* homologue) was localized in the genome of the cnidarian *N*. *vectensis* using tBLASTn-alignment (Fig 1). Sequences with homology to LDL receptor, immunoglobulin, laminin B and G chains, and EGF were found in an order identical to that of domains II, III, IV, and V of perlecan in bilaterians (Fig 1). No Sperm protein, Enterokinase, and Agrin (SEA—domain I) homologue or O-glycosylation sites were found upstream of *N*. *vectensis perl* domain II. This finding prompted the search for an *HSPG2* homologue in the genome of the seemingly simpler species *T*. *adhaerens*.

### *T*. *adhaerens perl* is split into two separately transcribed genes

5’ RACE PCR reveals that *T*. *adhaerens perl* is transcribed as two separate genes (Fig 1), which although only thirteen kilobase pairs apart on the chromosome, have maintained separate promoters in this organism. There are two likely start sites of transcription for both *T*. *adhaerens perl 1* and *2* revealed by 5’ RACE PCR. All efforts to connect these two genes by PCR-based methods were fruitless, indicating that these two products are transcribed separately. The two separate *T*. *adhaerens* transcripts corresponding to perlecan are most likely to originate from separate genes.

### *Perl* assembly is not conserved among *Amphimedon queenslandica*, *Mnemiopsis leidyi* or *Monosiga brevicollis*

Scattered perlecan-like motifs were identified by tBLASTn-alignment in the genome of *A*. *queenslandica* and in the transcriptomes of various other poriferan species (Fig 1). Genes encoding numerous IgG repeats are present in the *A*. *queenslandica* genome, as well as multiple LDLR modules and laminin motifs. These sequences often are clustered together in groups of multiple repeats. The regions encoding the would-be domains of *perl* are encoded on separate scaffolds. These domains are encoded by distant genes (at minimum 2,500 kilobase pairs), if not on different chromosomes. The starting point for intronic recombination-based formation of a unified *perl* gene appears to the region rich in Ig modules, where there is one Ig encoded 44 kb upstream of a region of 37 predicted Ig modules in close proximity. This spacing is similar to that in *T*. *adhaerens perl*, where domain II houses one Ig module, and then 12 kb downstream are encoded 38 tandem Ig modules, longer than the human Ig encoding region of *HSPG2*.

Whereas there is no evidence of a *perl*-like gene in the *A*. *queenslandica* genome, the transcriptomes of the homoscleromorph sponge *Corticium candelabrum* and *Sycon coactum* (of the order Leucosolenia) each encode gene products that are similar to some parts of *T*. *adhaerens perl II* in structure (Fig 1). LDLR and Laminin B modules are encoded by the genomes of these organisms, but they are scattered and not grouped into one gene assembly as in *perl*.

Very few perlecan-like motifs were identified by tBLASTn-alignment in the transcriptome of the ctenophores *Mnemiopsis leidyi* and *Pleurobrachia bachei* or the genome of the unicellular choanoflagellate *Monosiga brevicollis*. *M*. *leidyi* and *P*. *bachei* are part of the ctenophore family which, as recently described [24], evolved separately from the rest of the metazoan tree. Human perlecan protein modules BLASTed to 132 separate *M*. *leidyi* transcripts, but most of these only included one type of folding module. Some hits included multiple folding motifs as shown in Fig 1, but none of these transcripts encode perlecan homologues. Homologues for several laminin chains, fibulin, Notch, protocadherin, attractin and neurexin were found encoding various types of perlecan-like modules. The *P*. *bachei* genome encodes various laminin, LDLR, hemicentin, sortilin and semaphoring homologues but no perlecan-like gene [24]. Our analysis of the *M*. *brevicollis* genome found only one perlecan-like IgG module, three LDLR modules, and four laminin EGF-like modules on different scaffolds (Fig 1). There is no single gene encoding a perlecan-like protein in ctenophores or choanoflagellates. The recently published genome of the filasterian *Capsaspora owczarzaki* also contains no *perl* homologue [25].

### *HSPG2*/*perlecan* and the evolution of basal metazoans

**Fig 2:**
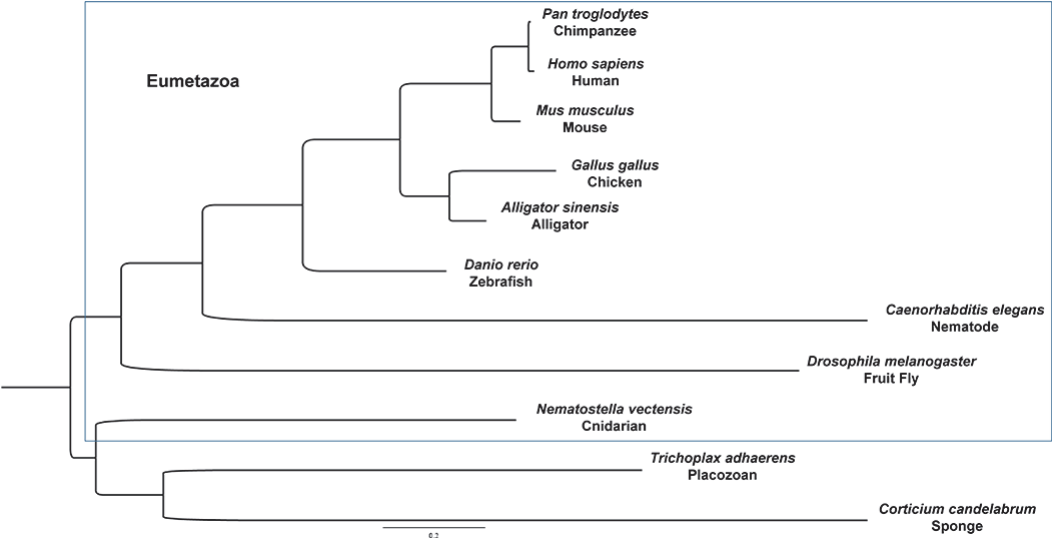
Molecular phylogenetic analysis by maximum likelihood method. The evolutionary history of perlecan was inferred by using the Maximum Likelihood method. Branch lengths are calculated according to nucleic acid substitutions per site. The eumetazoans are delineated by a dotted line.

Fig 2 shows a phylogenetic tree produced using the peptide sequence of representative perlecan homologues and the Ig-laminin G module-containing sequence of *C*. *candelabrum* to show that these peptide sequences support the conventional evolutionary paradigm. The phylogenetic analysis is consistent with *T*. *adhaerens* and *N*. *vectensis* being basal to bilaterians, and derived from some other common ancestor. As additional evidence of the accuracy of our analysis, the relationships amongst other perlecan peptide sequences displayed here follow the evolutionary paradigm for the animal kingdom. Comparisons between perlecan of *H*. *sapiens* and *T*. *adhaerens* may reveal evolutionary significance of various amino acid residues and motifs.

### Domain IV of *T*. *adhaerens* perl 1 and 2 as compared to that of *H*. *sapiens* perlecan

**Fig 3:**
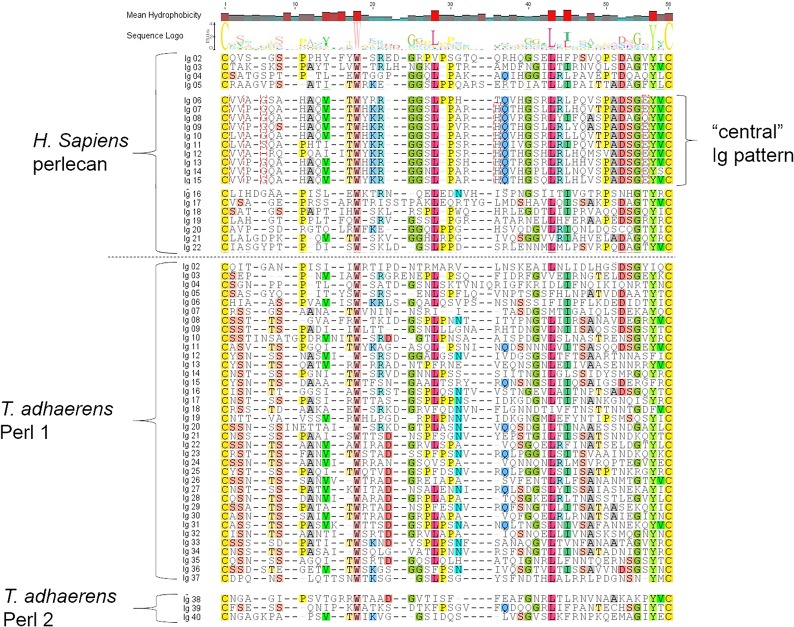
Comparison of *H*. *sapiens* perlecan domain IV and *T*. *adhaerens* perl 1 and perl 2 domain IV. The primary structure of domain IV Ig modules were aligned, including all sequences between disulfide-bonded cysteines in each Ig module. The sequences were aligned and residues conserved in 25% or more of these Ig sequences were highlighted. The central Ig modules of *H*. *sapiens* domain IV, distinct from the outer Ig modules, are shown separately. Residues of the central Ig modules that are conserved amongst *H*. *sapiens* domain IV Ig modules, but not when compared alongside those of *T*. *adhaerens*, are outlined with a red dashed line. Ig sequences originating from *T*. *adhaerens* perl 1 and perl 2 are shown separately.

The domain IV of perlecan is the largest part of the protein. We performed alignments comparing domain IV Ig modules in *H*. *sapiens* and *T*. *adhaerens* to study the conservation of amino acid residues in this large domain. Domain IV of *H*. *sapiens* perlecan includes 21 Ig modules that, including the single Ig module of domain II, add up to 22 total Ig modules throughout the protein (Fig 3). We previously have speculated that ten of the central Ig modules of human perlecan domain IV were inserted into domain IV via intronic recombination after formation of the gene [1]. These central modules display a distinct sequence pattern that is not conserved in the outer modules. *T*. *adhaerens* perl 1 and 2 include 40 Ig modules, making this the longest identified domain IV of any perlecan homologue studied to date (Fig 3). Although this perlecan homologue encodes more Ig modules, there is no apparent segregation of a distinct folding pattern of the central Ig modules compared to the outer Ig modules. In fact none of the distinctive motifs common to the central Ig modules of human perlecan can be found. Several highly conserved residues *between H*. *sapiens* and *T*. *adhaerens* perlecan include a tryptophan, two leucines and a tyrosine, which may betray their functional significance in the formation of the common Ig fold (S1 Fig). To study the conservation of perlecan’s core protein function in basal metazoans, we performed expression pattern analysis and tissue regeneration assays using *N*. *vectensis* as a native model system that lacks the N-terminal SEA domain.

### *N*. *vectensis perl* encoded transcript is expressed in the ectoderm of the scapus and the ectoderm of the mesentery, but not in the ectoderm of the tentacles

**Fig 4:**
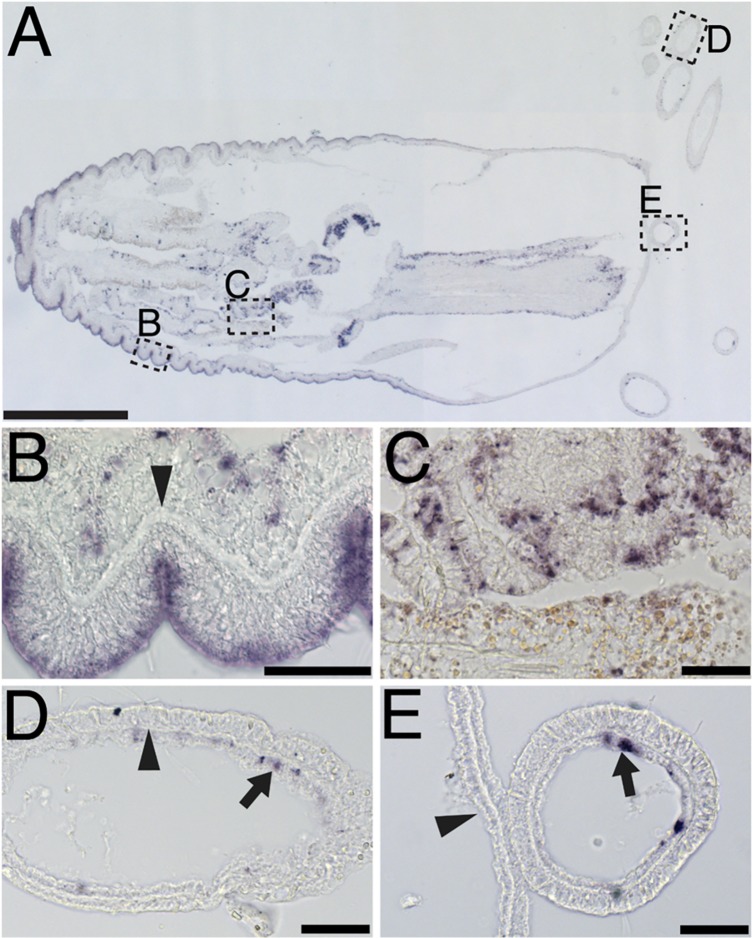
Perlecan is expressed in the ectoderm of the scapus, irregularly in the mesentery, and irregularly in the inner cell layer of the tentacles. An adult *N*. *vectensis* (A) was tested for *perl* mRNA expression. The ectoderm of the body wall has consistent *perl* mRNA signal in the ectoderm, whereas the gastroderm is mostly negative for *perl* mRNA. The acellular mesoglea is clearly visible in the physus and tentacle (arrowheads, B, D). The mesentery expresses *perl* mRNA in a sporadic fashion, with pigmented cells not expressing *perl* (C). Tentacle cells only express *perl* sporadically in the gastroderm (arrows, D,E). The oral end of the scapus does not express the *perl* transcript (A, arrowhead, E). Scale bars, A—0.5mm, B-E—40 μm.

*In-situ* hybridization of *N*. *vectensis perl* reveals that the transcript is expressed throughout the ectoderm of the scapus and physus, as well as in select cells of the mesentery (Fig 4A). Although there is a basal lamina of the gastroderm, there is little staining for the *perl* transcript in the gastroderm of the scapus. The acellular mesoglea is evident in high magnification images of the scapus (Fig 4B), where it appears that the ectoderm expressed the *perl* transcript. *Perl* expression in the mesentery is mosaic in pattern of appearance (Fig 4C). Little staining is seen in the ectoderm of the tentacles, but staining is evident in the gastroderm of the tentacles, reflecting the expression pattern of the mesoderm of the scapus (Fig 4D and 4E). *Perl* expression in the scapus appears to be polarized towards the physus, with the oral structures and tentacles only sporadically expressing *perl* (Fig 4A, 4D and 4E).

### During *N*. *vectensis* regeneration, *perl* expression is activated at stage 2.5 in the regenerating pharynx and tentacles

**Fig 5:**
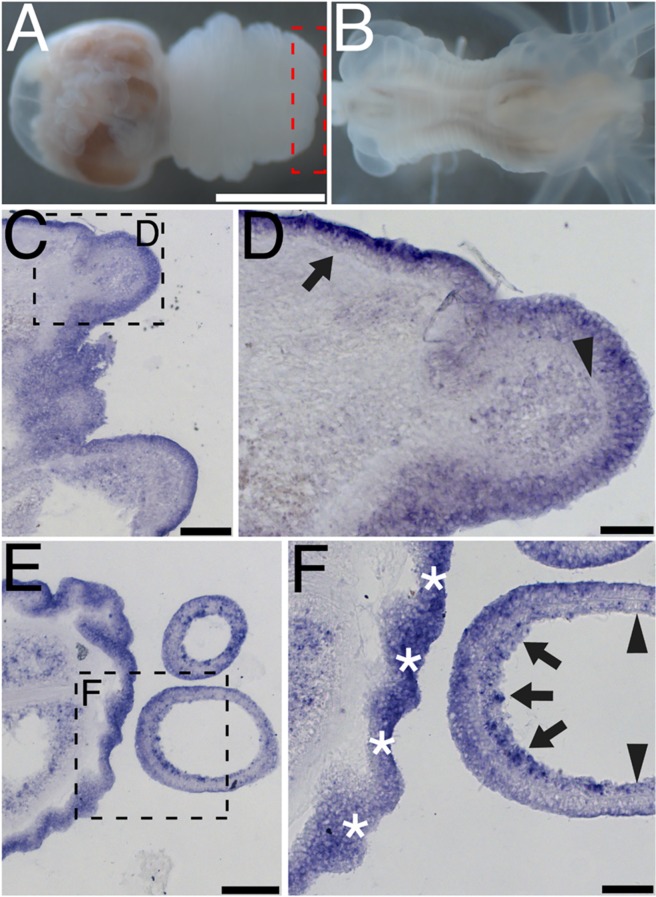
Perlecan is expressed in the regenerating oral region and tentacles at stage 2.5 through stage 4. Animals were bisected midway through the mesentery region. Representative animals are pictured 48 hours post-bisection in (physal end-A, oral end-B). Regeneration of the oral structures and tentacles was followed in the area of the red box in (A). *Perl* activation is visible during regenerative stage 2.5–3 in the tentacle bud and oral structures (C). The developing cellular boundary between ectoderm and gastroderm is visible in the primordial tentacle (arrowhead, D) and is distinct from the developed mesoglea in the regenerated oral pole of the scapus (arrow, D). *Perl* remains elevated in the tentacles and oral structures during the intermediate stage of tentacle regeneration (stage 4, E, F). *Perl* expression recedes in the tentacles as they develop mesoglea (arrowheads, F) and remains where the mesoglea is not yet fully visible (F, arrows). Prior to completion of regeneration, *perl* expression continues in the oral ectoderm (asterisks, F). Scale bars, A,B-500μm C,E-100 μm D,F-40μm.

*Perl* mRNA expression during *N*. *vectensis* regeneration was studied using *in-situ* hybridization (Fig 5). Organisms were fixed and sectioned at stages one through five of the regenerative process. At stages one and two, *N*. *vectensis perl* mRNA is expressed much as it is in the intact organism, with expression of *perl* throughout the ectoderm. mRNA expression is seen in the ectoderm of the physus and scapus during stages one and two, as well as in individual cells in the mesentery that are retained during bisection of the animal (data not shown). At stage 2.5–3, activated tissue is evident as the oral end of the organism, and *de novo N*. *vectensis perl* mRNA signal is visible (Fig 5C and 5D). Tentacle bud cells transcribe *N*. *vectensis perl* mRNA at this stage, and are forming an acellular layer between the developing ectoderm and gastroderm (Fig 5D, arrowhead). The primordial gastroderm forms what appears to be an inner cell mass in the developing tentacle and also expresses *perl*. At stage 4, tentacles are elongating and, in contrast to undamaged tissue, *perl* mRNA is resident in the tentacle ectoderm (Fig 5E and 5F). The more central regions of the developing tentacle lose *perl* expression as a more well-defined ectoderm and gastroderm become distinct during regeneration along with a visible acellular mesoglea (Fig 5F, arrowheads). However, the proximal side of the developing tentacle exhibits incompletely developed ectoderm and gastroderm layers with relatively high levels of *perl* expression and has yet to develop an acellular mesoglea. The ectoderm of the oral structure of the physus also expresses *perl* at stages 2.5–4 (Fig 5C and 5E), in contrast to oral ectoderm of the adult *N*. *vectensis* (Fig 4A). Expression of *perl* during regeneration was verified by qPCR and demonstrated a peak of expression at 6 days post-wounding, roughly during stage 2.5–4 of regeneration (S2 Fig). After completion of regeneration, *perl* expression mirrors that of the normal adult *N*. *vectensis* (data not shown).

## Discussion

In the most parsimonious evolutionary scenario, the common ancestor of cnidarians, placozoans and bilaterians possessed two genes that fused to form HSPG2 on the lineage leading to the common ancestor of bilaterians. In *T*. *adhaerens*, the *HSPG2* orthologs exist on the chromosome in nearly the same arrangement as in bilaterians, but are split into two genes. *T*. *adhaerens perl* encodes an SEA domain, which distinguishes this gene and its product from *D*. *melanogaster trol* [6], *C*. *elegans unc-52* [5], neither of which are predicted to contain an SEA fold. The SEA domain has a regulatory activity on glycosylation during post-translational modification [26]. Unlike mammalian *HSPG2*, *T*. *adhaerens perl* and *N*. *vectensis perl* do not encode consensus GAG attachment sites in the N-terminus, although the *T*. *adhaerens* genome does encode genes with homology to the heparan sulfate synthetic machinery (S1 Table), similar to those reported to function in *N. vectensis [27]*. The various folding domains of *T*. *adhaerens perl* are organized in the same N-terminus to C-terminus order as perlecan of bilaterians. At least 38 Ig folds exist between the domain IV orthologues of *T*. *adhaerens perl 1* and *perl 2*, 17 more than the 21 encoded by domain IV of the human gene, and more than the most found in any *HSPG2* orthologue in the vertebrate tree. It is a straightforward deduction that intronic recombination between *T*. *adhaerens perl 1* and *perl 2* could have created one unified gene expressing close to 21 Ig folds in domain IV. A less parsimonious scenario that cannot be ruled out with current data is that a single gene existed in the common ancestor of bilaterians and placozoans, with a subsequent gene fission event on the T. adhaerens lineage.

Exon shuffling or intronic recombination events were key to the evolution of ECM and membrane proteins [28]. As discussed in previous publications and included in Fig 1, the *A*. *queenslandica* genome contains genes encoding orthologues of all of the constituent pieces of the perlecan protein, but they are scattered throughout the genome. A single region of the *A*. *queenslandica* genome that houses nearly 40 repeats homologous to Ig modules is present, and it is possible that this, in addition to the other regions encoding homologous modules, is the site of origin of the *T*. *adhaerens perl* gene. Our analysis of the *T*. *adhaerens perl* indicates that nearly every piece of the gene could have been assembled from exons of other previously extant homologous genes. It is also possible that this is the remnant of a *perl* gene which existed in the common ancestor of sponges and placozoa.

Phylogenetic analysis of *HSPG2/perl* transcripts reveal that the *T*. *adhaerens* and *N*. *vectensis perl* are most closely related to one another, in agreement with the commonly accepted view of the basal metazoans’ place on the metazoan tree. The core observation of this analysis is that *T*. *adhaerens perl* is a good proxy for the ancestral *perl*. Although one study found [29] that a perlecan homologue is found in the publically accessible genome of the hemoscleromorph sponge *Oscarella carmela*, our search for this homologue found many laminin, LDL receptor, Titin, and hemicentin homologues, but only one curious transcript that encoded a protein identified by BLASTp as a match for *HSPG2* (S2 Fig). This *O*. *carmela HSPG2* homologue’s protein product differs greatly from perlecan, including von Willebrand A modules interspersed amongst only five Ig modules and four LDLRa modules. Because no other significant matches were identified by BLAST, this transcript and putative protein product most likely are unique to sponges. Another study has suggested that the demosponges such as *A*. *queenslandica* have diverged from the rest of the animal kingdom more so than the homoscleromorph sponges [30]. Our finding of a *perl* II-like transcript in the transcriptomes of sponges *C*. *candelabrum* and *S*. *coactum* supports this notion.

We propose that the assembled *perl* gene first appeared in the common ancestor of placozoans, cnidarians and bilaterians, not in the common ancestor of all metazoans. The presence of *perl* complicates previously published conclusions illustrating by electron microscopy the apparent lack of an organized ECM in placozoan tissue, but recent proteomic data supports our findings, in that perlecan-like peptides are found in the placozoan [31]. It is thought that *M*. *brevicollis* secretome serves to mediate interactions between the organism and its environment [11]. Similarly, it is possible that the slime layer of *T*. *adhaerens* evident on the upper epithelium comprises the bulk of their secreted proteins. If the *T*. *adhaerens* perlecan is secreted apically instead of basally, this would explain the lack of visible ECM in electron microscopic imaging. In this scenario, the interior movement of secreted perlecan to the true basement membrane in metazoans would be a later refinement of eumetazoans.

Perlecan in vertebrate animals is critical for proper development and maintenance of tissue architecture [4]. In addition the protein is deposited during the later wound healing phase of various model systems [32]. We hypothesized that the protein is needed to ensure proper tissue architecture during regrowth of *N*. *vectensis*, and the gene no longer is transcribed actively after reformation of the mesoglea and clear differentiation of the ectoderm and gastroderm cell layers. Other studies have demonstrated upregulation of various genes during regeneration [33], but this is the first to study the expression of a basement membrane component during this process. During *N*. *vectensis* regeneration, an increase in expression of tissue inhibitors of metalloproteinases (TIMPs) directly surrounding the wounding site is observed [33], which would allow for the secretion and integration of new extracellular matrix components, including *perl*, during the regeneration of distinct tissue layers and the acellular mesoglea. In both Hydra and *N*. *vectensis*, a quiescent stem cell population proliferates during regeneration in order to form functional head structures [14,34]. This proliferative phase must precede *perl* expression and regeneration of the mature mesoglea, as we see upregulation of *perl* expression at stage 2.5 to stage 4, after head structures have reformed. Expression of *perl* at this stage is higher than that in intact adult *Nematostella* polyps (S2 Fig). Further, this role for *perl* in regeneration occurs in a species producing a protein that lacks the heparan sulfated growth factor binding domain I, hence we propose that the more C-terminal portions of the core protein play a key role in establishment of the barrier [1]. Addition of the SEA module and GAG-binding sites in human perlecan would thus be a later refinement to further support wound healing by providing a depot for growth factors coupled to the tissue barrier.

In summary, this study identified the evolutionary precursors of the human *HSPG2/perlecan* gene in the poriferan phylum and the *HSPG2*-like gene *perl* in the genome of the placozoan *T*. *adhaerens*. *Perl* is expressed in both placozoans and the cnidarian *N*. *vectensis*, and is activated during regeneration of lost tissue in the cnidarian. These findings provide paradigm changing insights on the evolution of this complex gene, most importantly pointing to its first appearance in the common ancestor of placozoans, cnidarians and bilaterians, but not in the metazoan ancestor as is often commonly thought. This insight deepens our understanding of the origins of animal multicellularity, extracellular matrix, and early tissue regeneration.

## Supporting Information

S1 Fig Three dimensional modeling of *T*. *adhaerens perl* Ig module protein folding. The publically accessible three dimensional protein structure modeling program PHYRE was used to predict the folding structure of *T*. *adhaerens perl* Ig modules. The sequence for a representative module is displayed (A) and the characteristic sheets of Beta strands are demonstrated in various rotations (B,C). Conserved residues are highlighted in yellow (C, D, E).
(TIF)

S2 Fig *Perl* expression reaches a peak at 6 days post-wounding. Whole polyps were collected at the indicated time points post-wounding. *Perl* transcript levels were compared to expression of *GAPDH*. Transcript levels are presented as fold change compared to zero days post-wounding. Three polyps were collected at each time point.
(TIF)

S3 Fig *HSPG2* homologues are absent from the *O*. *carmela* transcriptome. BLAST hits from the *O*. *carmela* transcriptome were translated and analyzed by PFAM (A). BLAST hit names were assigned by the transcriptome database. Under description is listed the protein homologue encoded by each transcript, what type of folding modules are included in this protein, and how many times each folding module is repeated in that protein. One protein was identified by BLAST as an *HSPG2* homologue, albeit with low confidence (red box—A). This protein is shown in schematic form in (B). Several Ig modules and LDL-receptor-like modules are encoded, as well as two von Willebrand A modules.
(TIF)

S1 Table Heparan sulfate biosynthetic genes of *T*. *adhaerens*. (TIF)
