# Supplementary figures and images for: Evolution of the Perlecan/HSPG2 Gene and Its Activation in Regenerating Nematostella vectensis
Source: PLoS One. 2015 Apr 15;10(4):e0124578. doi: 10.1371/journal.pone.0124578 (PMC4398486; doi:10.1371/journal.pone.0124578)

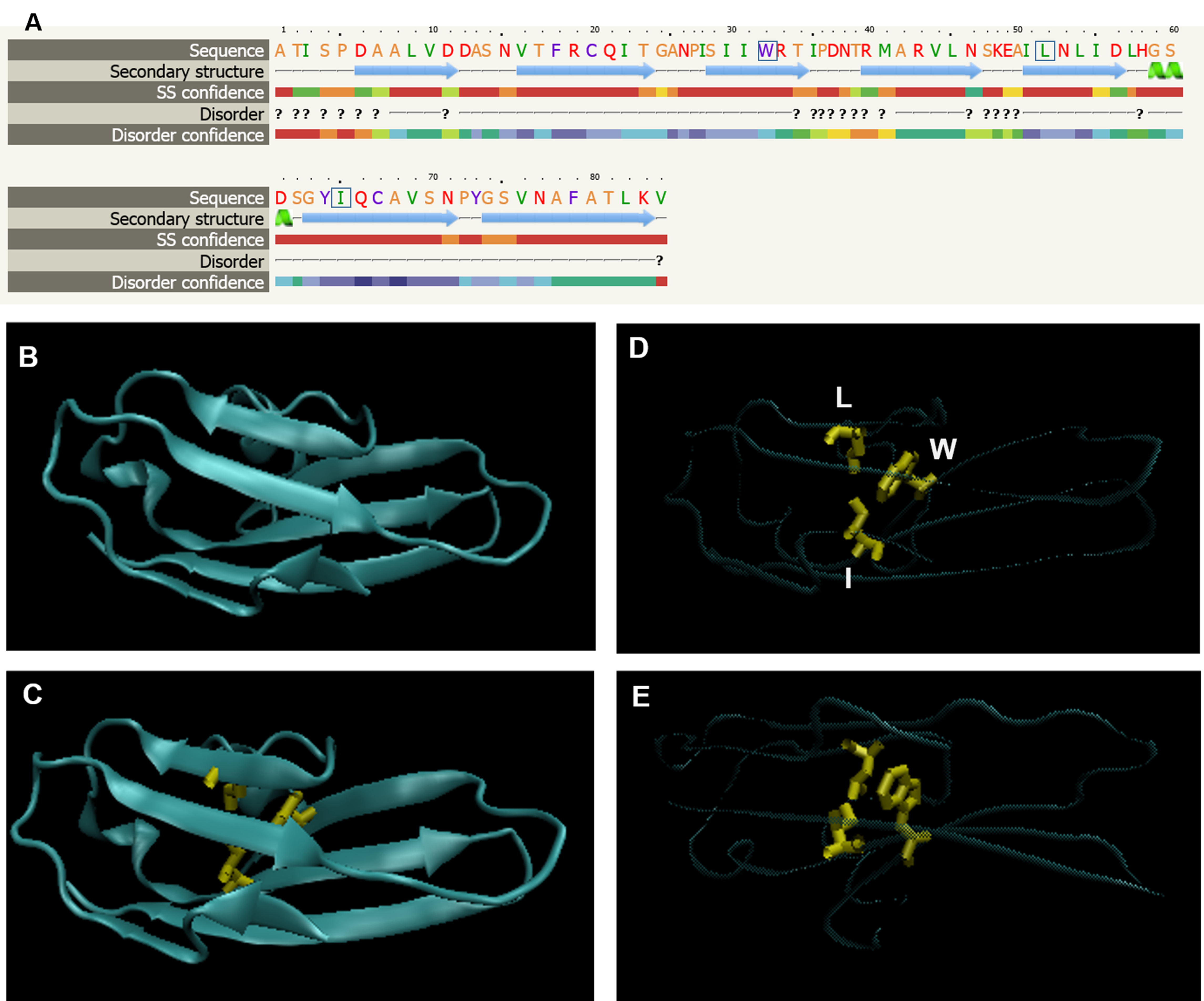

Supplement: S1 Fig — The publically accessible three dimensional protein structure modeling program PHYRE was used to predict the folding structure of T. adhaerens perl Ig modules. The sequence for a representative module is displayed (A) and the characteristic sheets of Beta strands are demonstrated in various rotations (B,C). Conserved residues are highlighted in yellow (C, D, E). (TIF) [file pone.0124578.s001.tif]

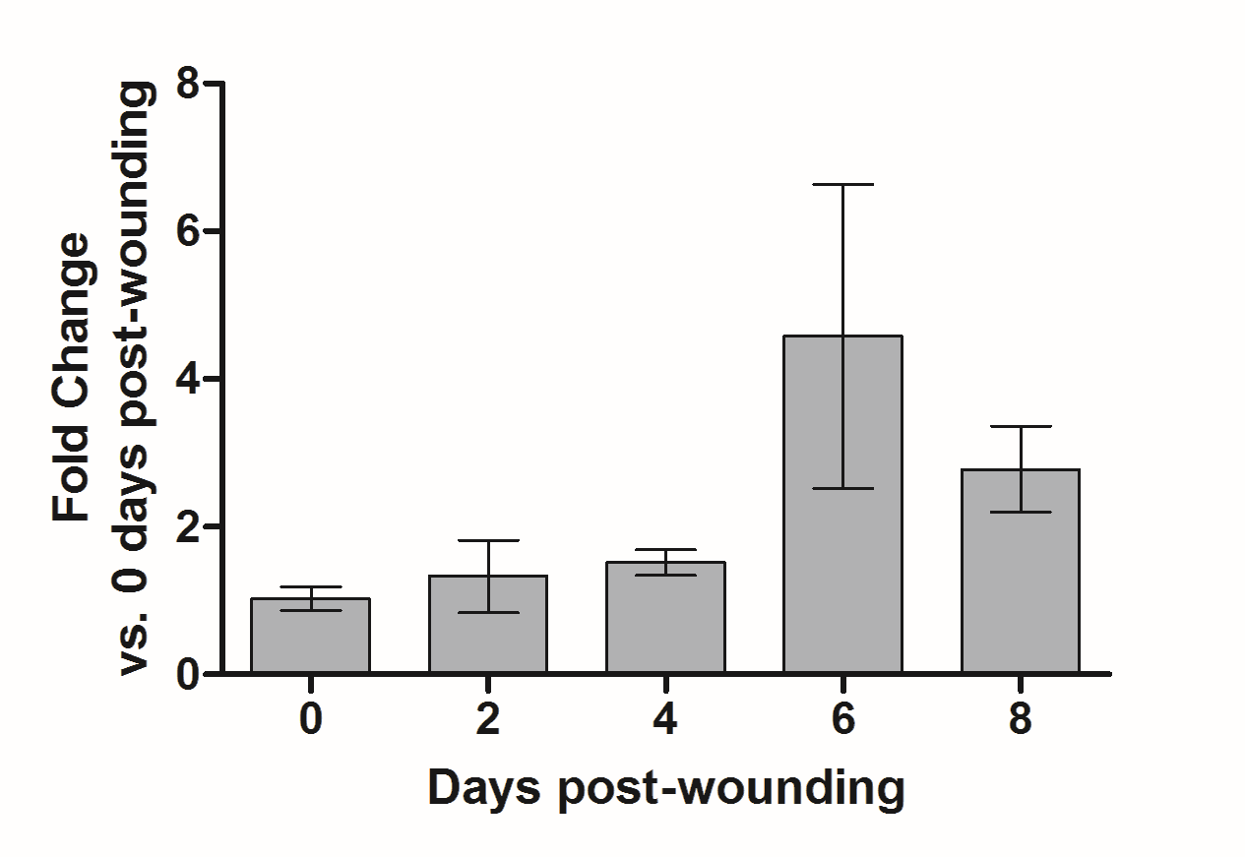

Supplement: S2 Fig — Whole polyps were collected at the indicated time points post-wounding. Perl transcript levels were compared to expression of GAPDH. Transcript levels are presented as fold change compared to zero days post-wounding. Three polyps were collected at each time point. (TIF) [file pone.0124578.s002.tif]

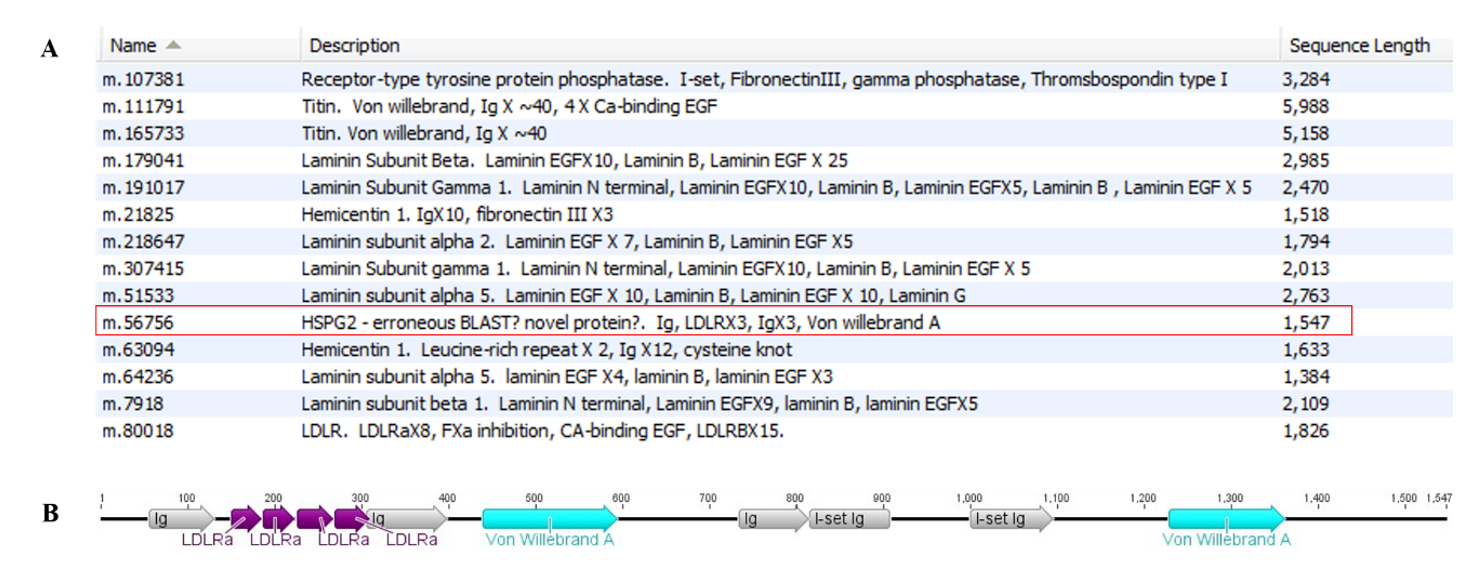

Supplement: S3 Fig — BLAST hits from the O. carmela transcriptome were translated and analyzed by PFAM (A). BLAST hit names were assigned by the transcriptome database. Under description is listed the protein homologue encoded by each transcript, what type of folding modules are included in this protein, and how many times each folding module is repeated in that protein. One protein was identified by BLAST as an HSPG2 homologue, albeit with low confidence (red box—A). This protein is shown in schematic form in (B). Several Ig modules and LDL-receptor-like modules are encoded, as well as two von Willebrand A modules. (TIF) [file pone.0124578.s003.tif]

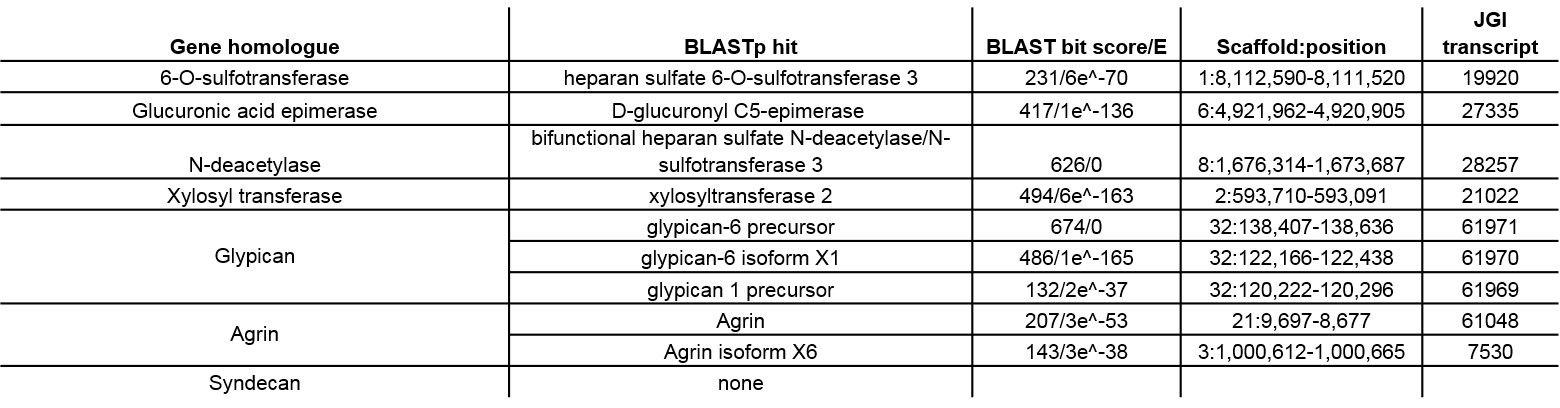

Supplement: S1 Table — (TIF) [file pone.0124578.s004.tif]
